# Supplementary figures and images for: Phototactic preference and its genetic basis in the planulae of the colonial Hydrozoan Hydractinia symbiolongicarpus
Source: bioRxiv. 2024 Apr 1:2024.03.28.585045. Preprint. [Version 1] doi: 10.1101/2024.03.28.585045 (PMC11014542; doi:10.1101/2024.03.28.585045)

A

MDS Plot for Count Data

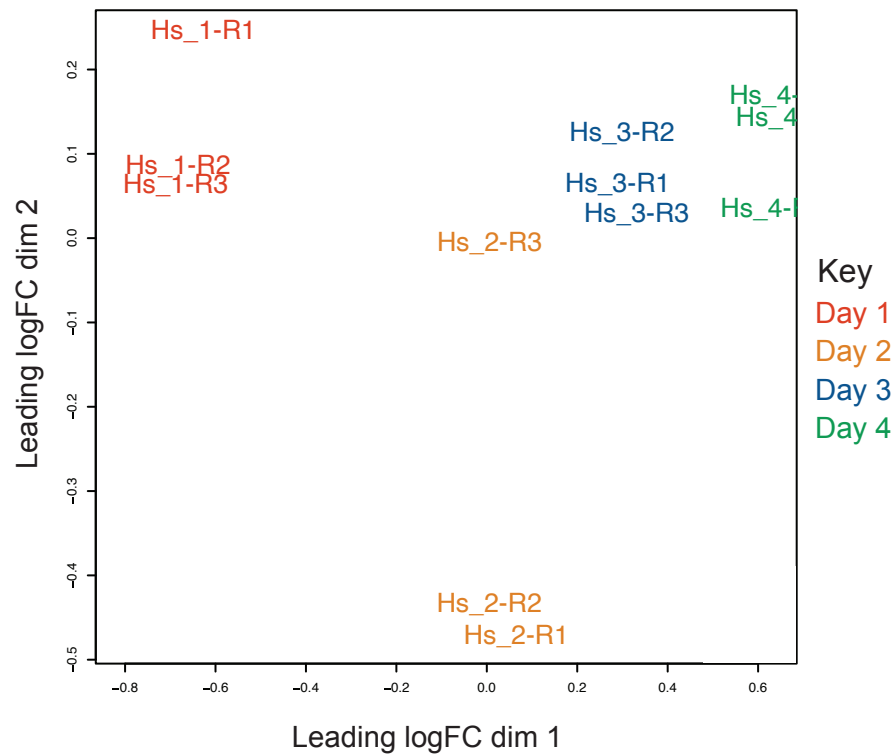

B

Total Number of Differentially Expressed Transcripts

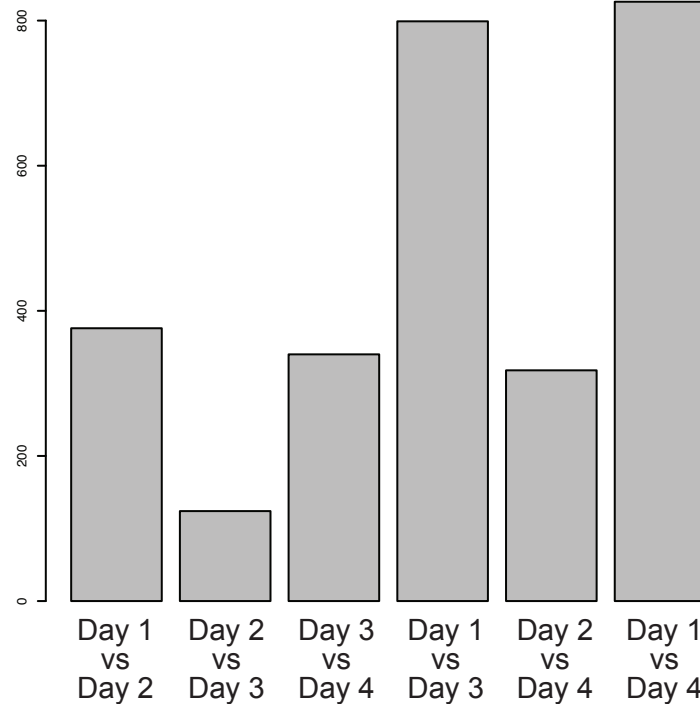

Supplement: Supplement 2 — Figure 2. Differential gene expression during planula development. (A) Multidimensional scaling (MDS) of 12 RNAseq replicates indicates clustering by developmental day. Red = day 1 (24hpf), yellow = day 2 (48hpf), blue = day 3 (72hpf), and green = day 4 (96hpf). (B) Pairwise comparison of the number of differentially expressed (DE) transcripts for each developmental day. Most DE transcripts occur between days 1 and 3, and days 1 and 4, while the lowest number of DE transcripts occur between days 2 and 3. [file media-2.zip › Fig_2.pdf]

A

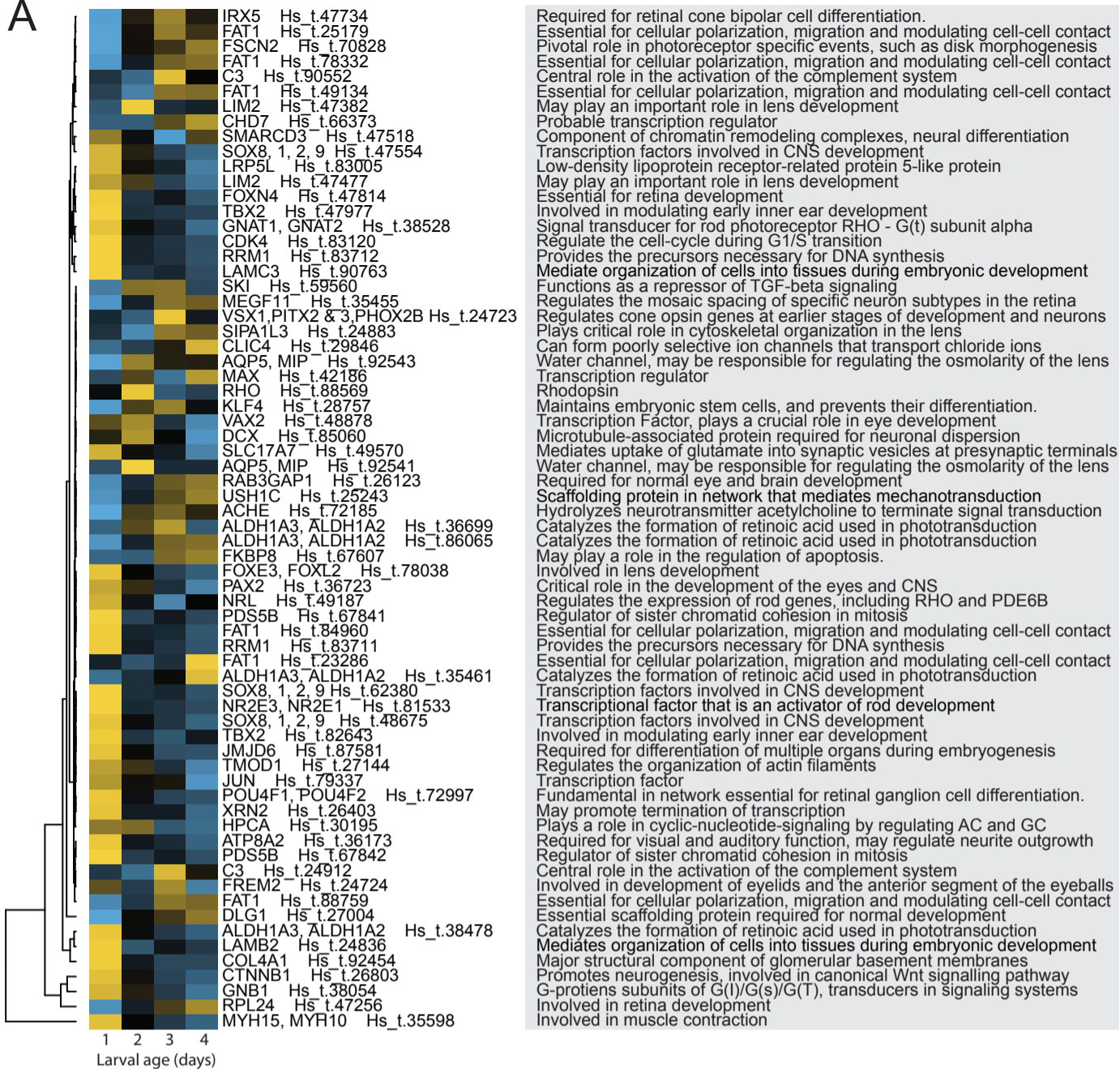

B

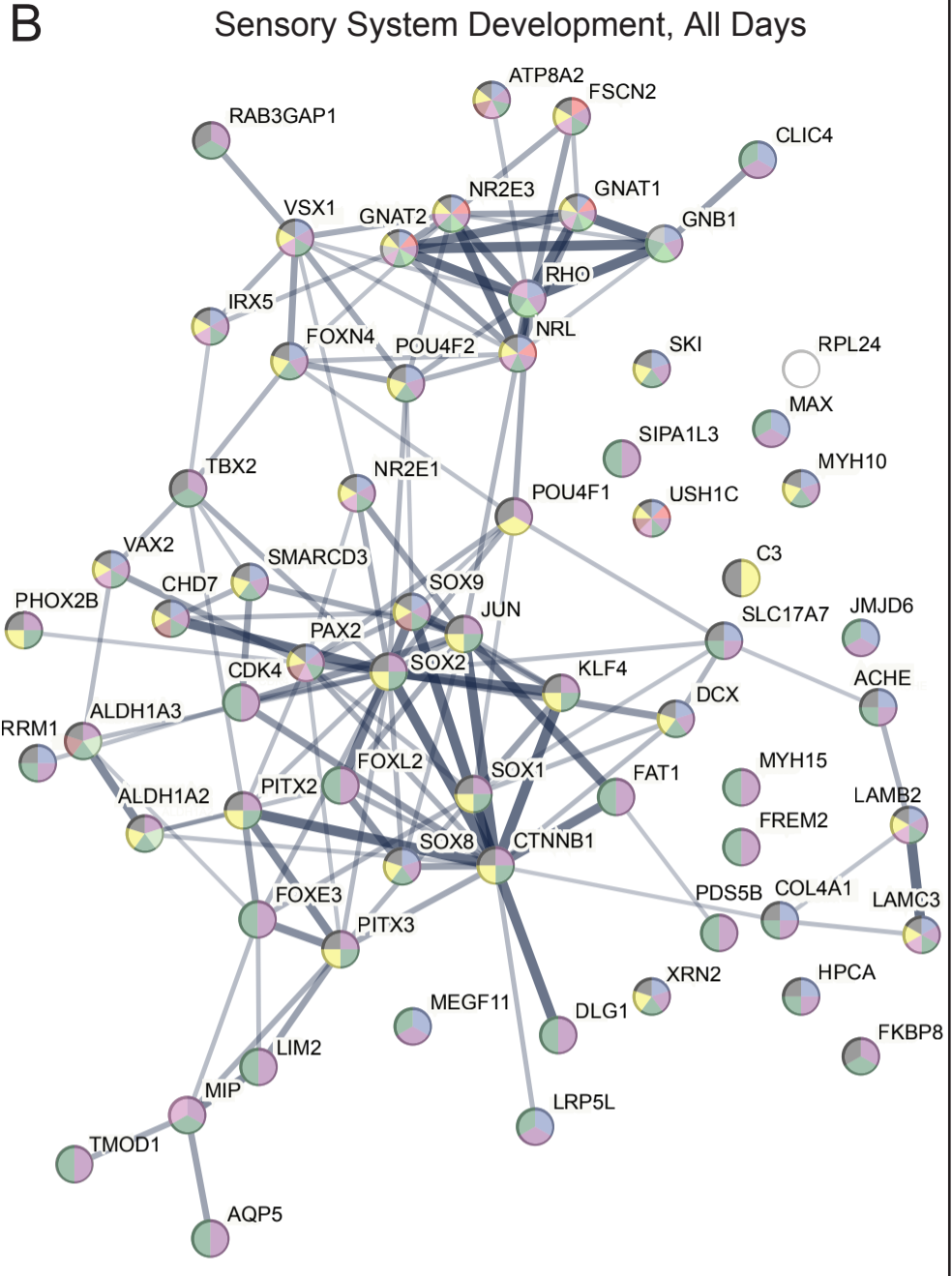

C

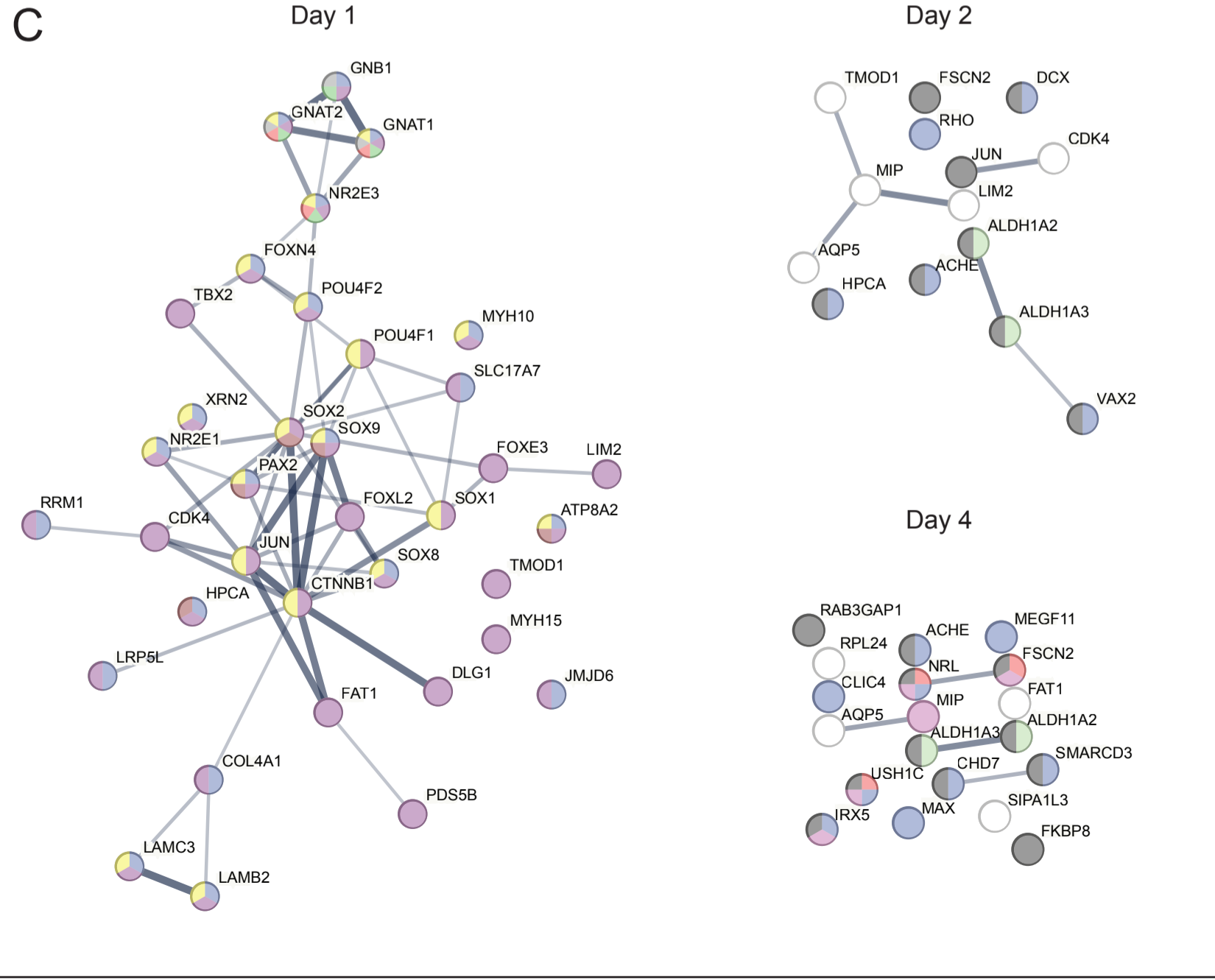

D

|          | #of nodes | # of edges | Expected # of edges | PPI enrichment p-value |
|----------|-----------|------------|---------------------|------------------------|
| All Days | 65        | 130        | 43                  | <1.0e-16***            |
| Day 1    | 36        | 57         | 21                  | 1.2e-10***             |
| Day 2    | 14        | 6          | 2                   | 0.0112**               |
| Day 3    | 25        | 10         | 4                   | 0.0113**               |
| Day 4    | 19        | 4          | 1                   | 0.0491*                |

Key

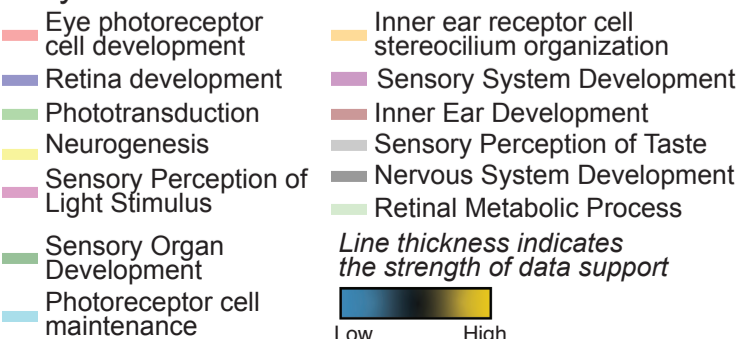

Supplement: Supplement 4 — Figure 4. Differential gene expression over development in the sensory system development gene set. (A) Differentially expressed genes over four days of development examining the sensory system development gene set derived from the GSEA (33). Each row is a single transcript labeled by the human gene symbols that are present in the same orthogroup as the planula transcript. High expression is yellow and low is light blue. Grey panels contain abbreviated descriptions of gene functions from UniProt (116). (B-D) STRING networks of significantly differentially expressed genes across four days of development where (B) contains all DE genes from all four days, and (C) contains subnetworks of DE genes by day. In each interaction map, line thickness indicates the strength of data support. Gene symbols are shaded by biological processes (Gene Ontology) or Annotated Keywords (UniProt). (D) Node and edge information for each network that includes the PPI enrichment p-values. In contrast to analyses based on the sensory perception of light stimulus gene set above, PPI enrichment p-values for the sensory system development gene set are highest in day 1, indicating greater functional interactions among genes expressed early in development. [file media-4.zip › Fig_4.pdf]

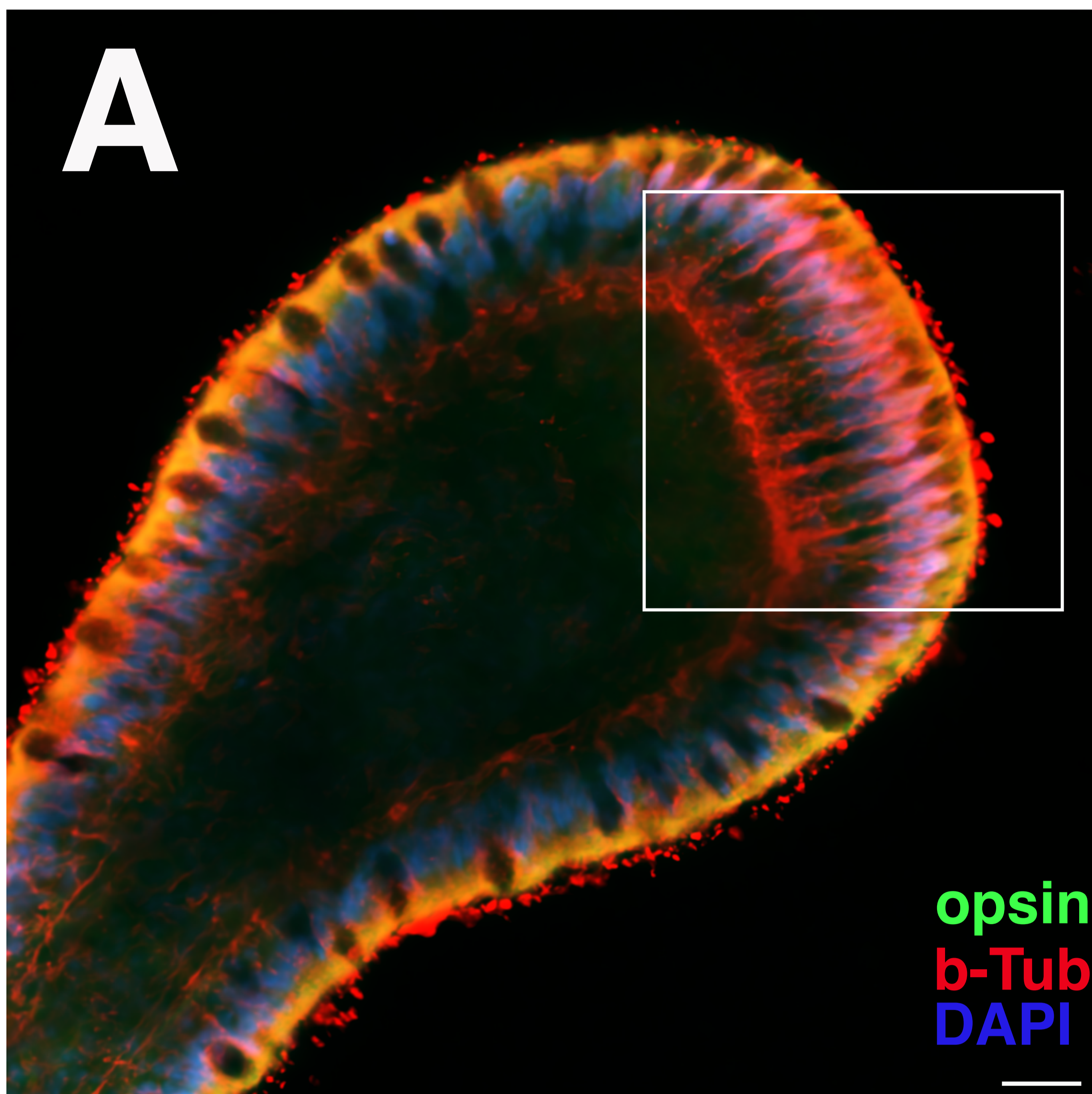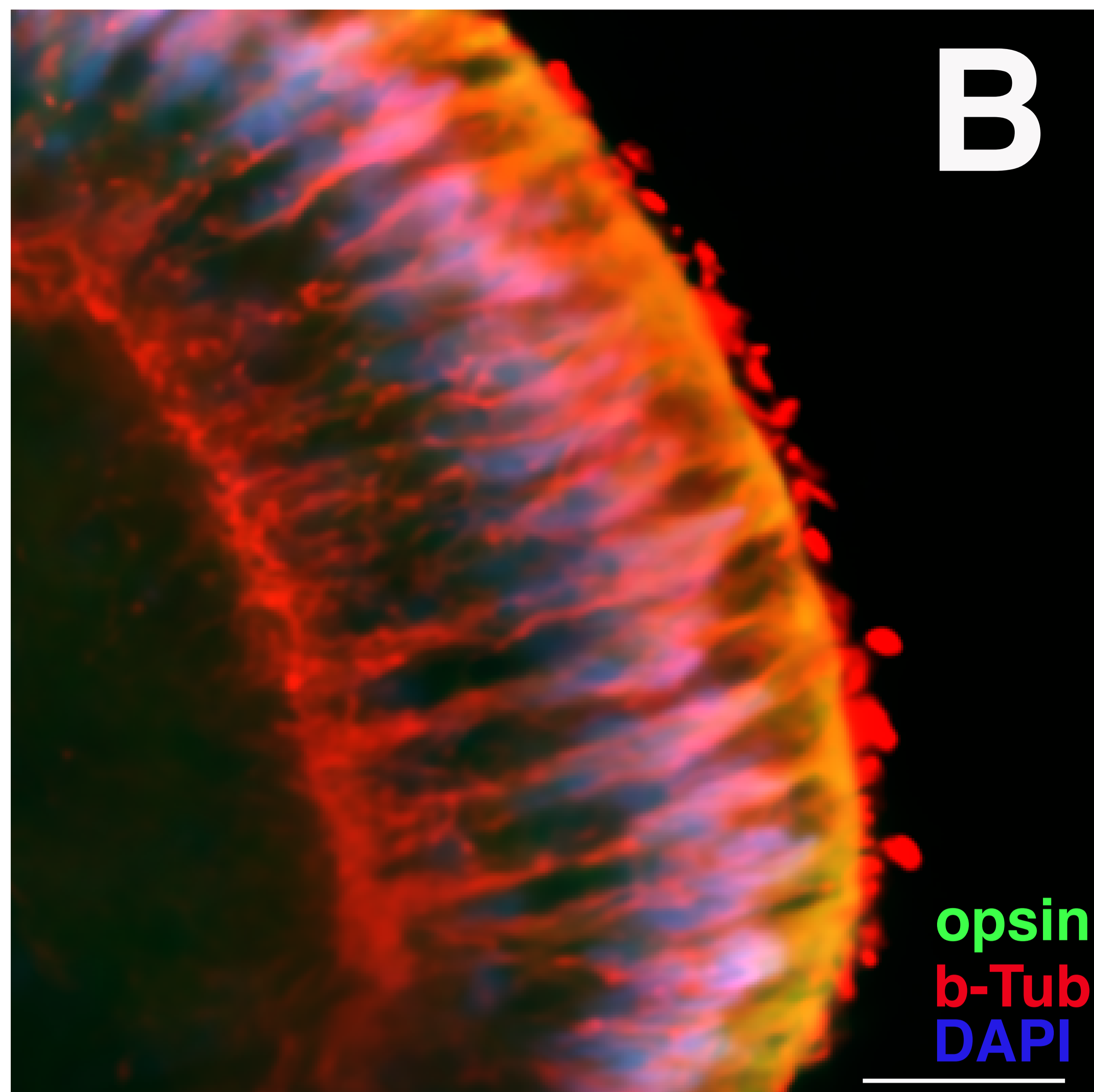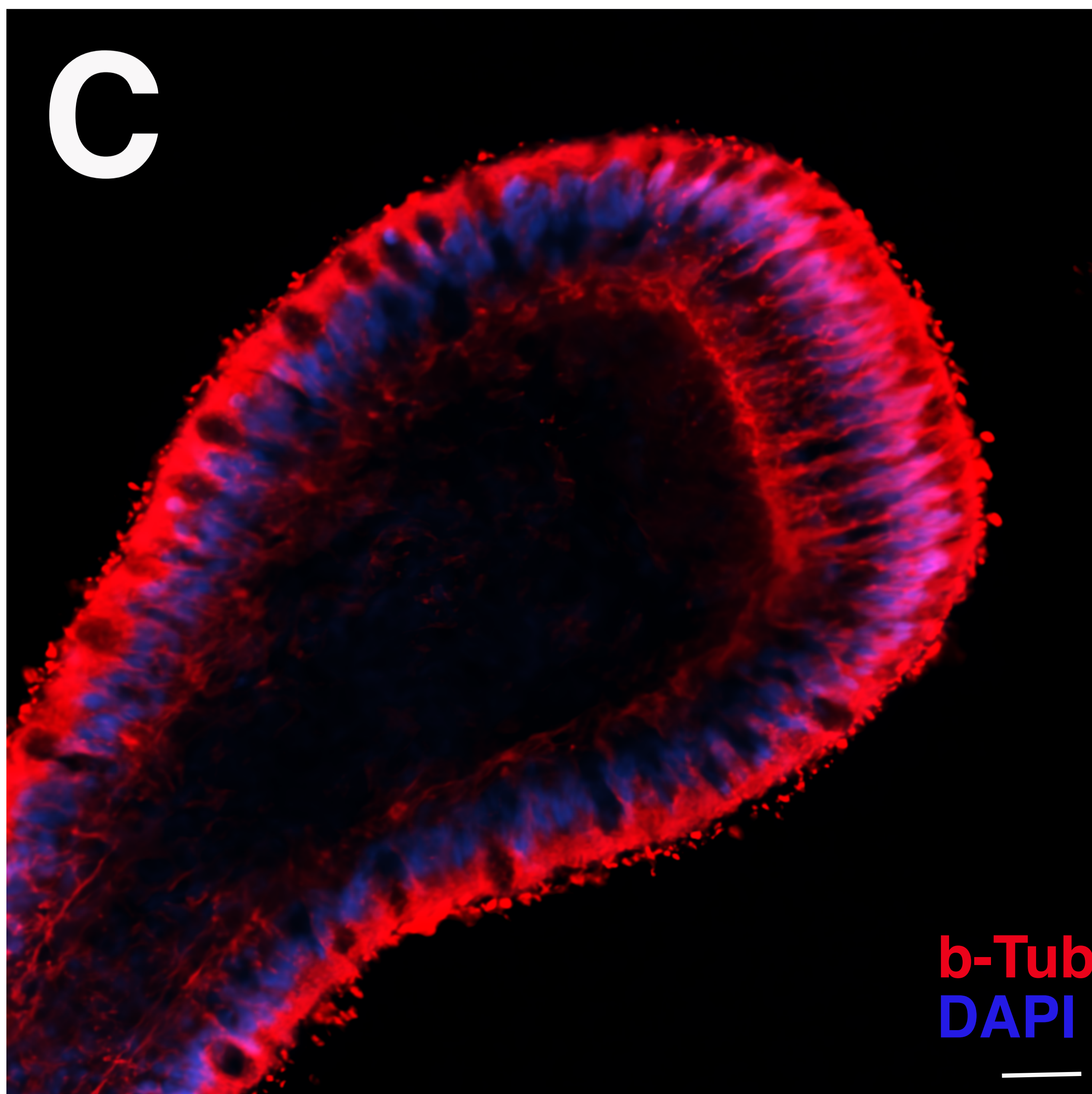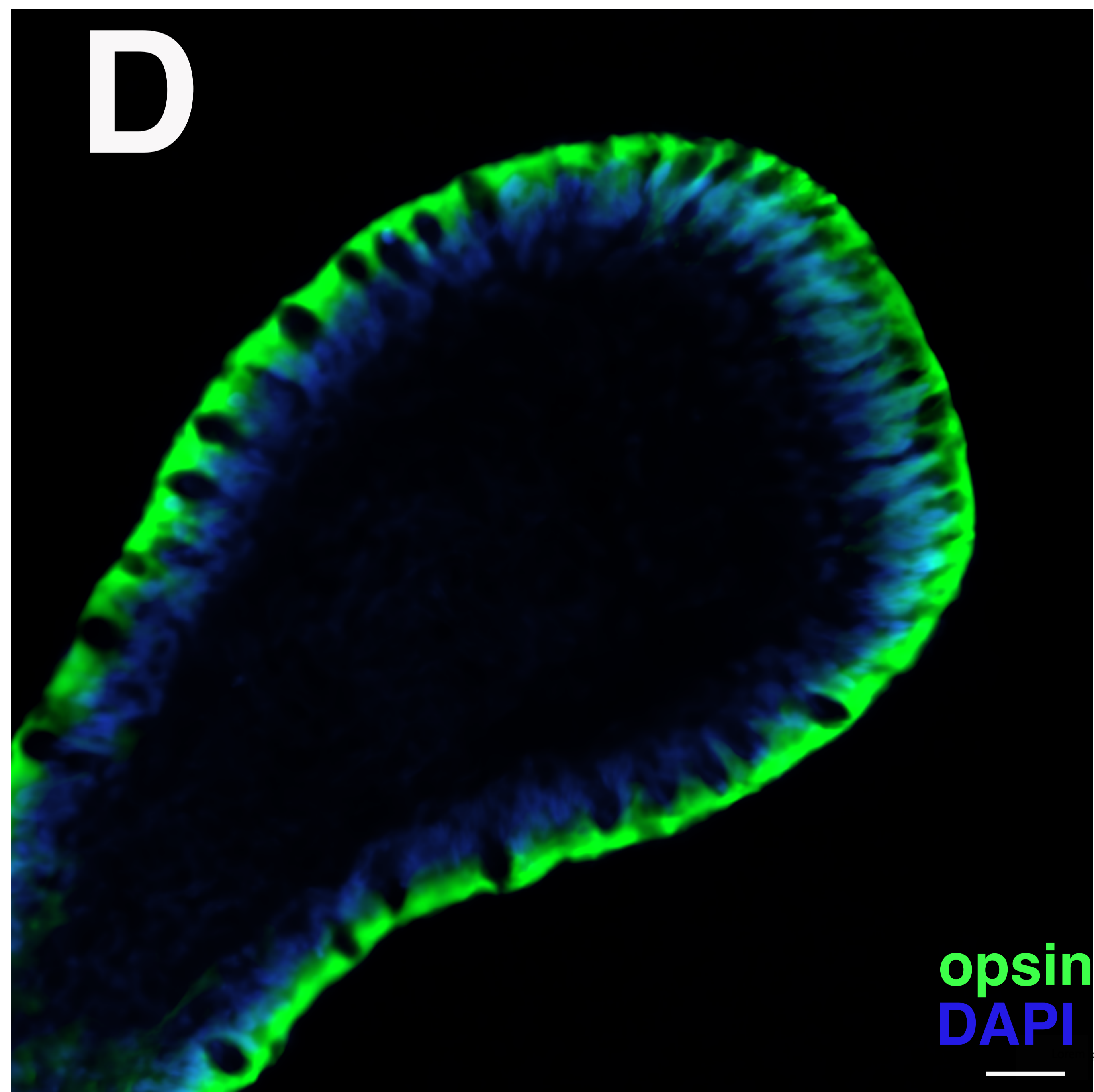

Supplement: Supplement 6 — Figure 6. Cnidopsin expressing bipolar neurons comprise the aboral neural plexus in H. symbiolongicarpus planula. (A-D) Day 3 (72hpf) larva labeled by RNA fluorescent in situ hybridization of cnidopsin (green; Hs_t.88569) combined with immunohistochemical staining of neural cells with anti-acetylated alpha-tubulin (red). Nuclear staining is by DAPI (blue). Box in (A) depicts section shown in panel (B) of the larval H. symbiolongicarpus neural plexus where opsin localizes to ciliary regions of bipolar sensory cells, which connect with ganglion neurons to form a plexus. Scale bars = 10um. [file media-6.zip › Fig_6.pdf]

Network of Genes that are Not Expressed in Sensory Perception of Light Stimulus Gene set

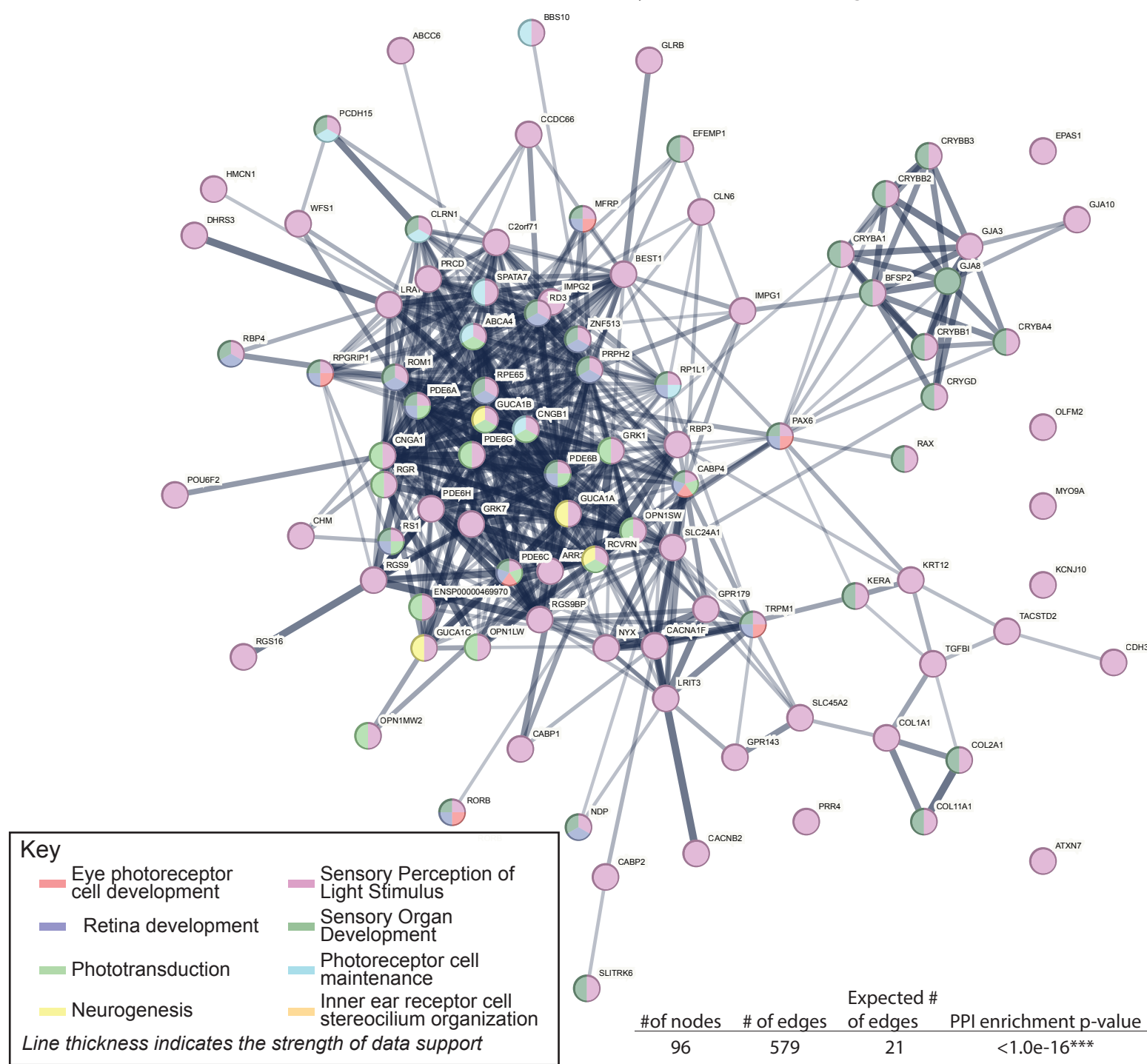

Supplement: Supplement 10 [file media-10.zip › Supp_Fig_2.pdf]

## RRH; OPN5; RHO; OPN4 OG0000063

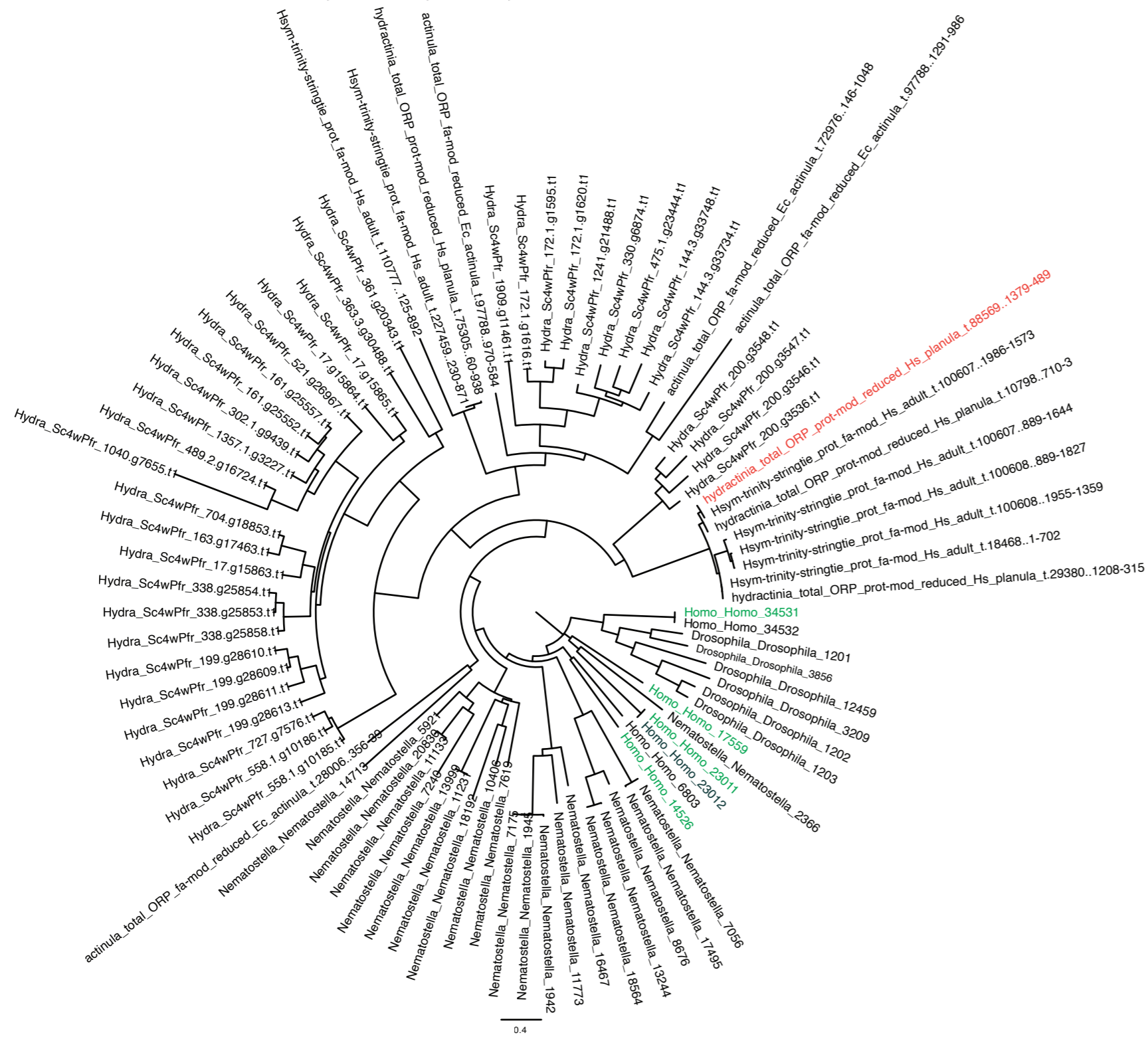

MIP OG0000218

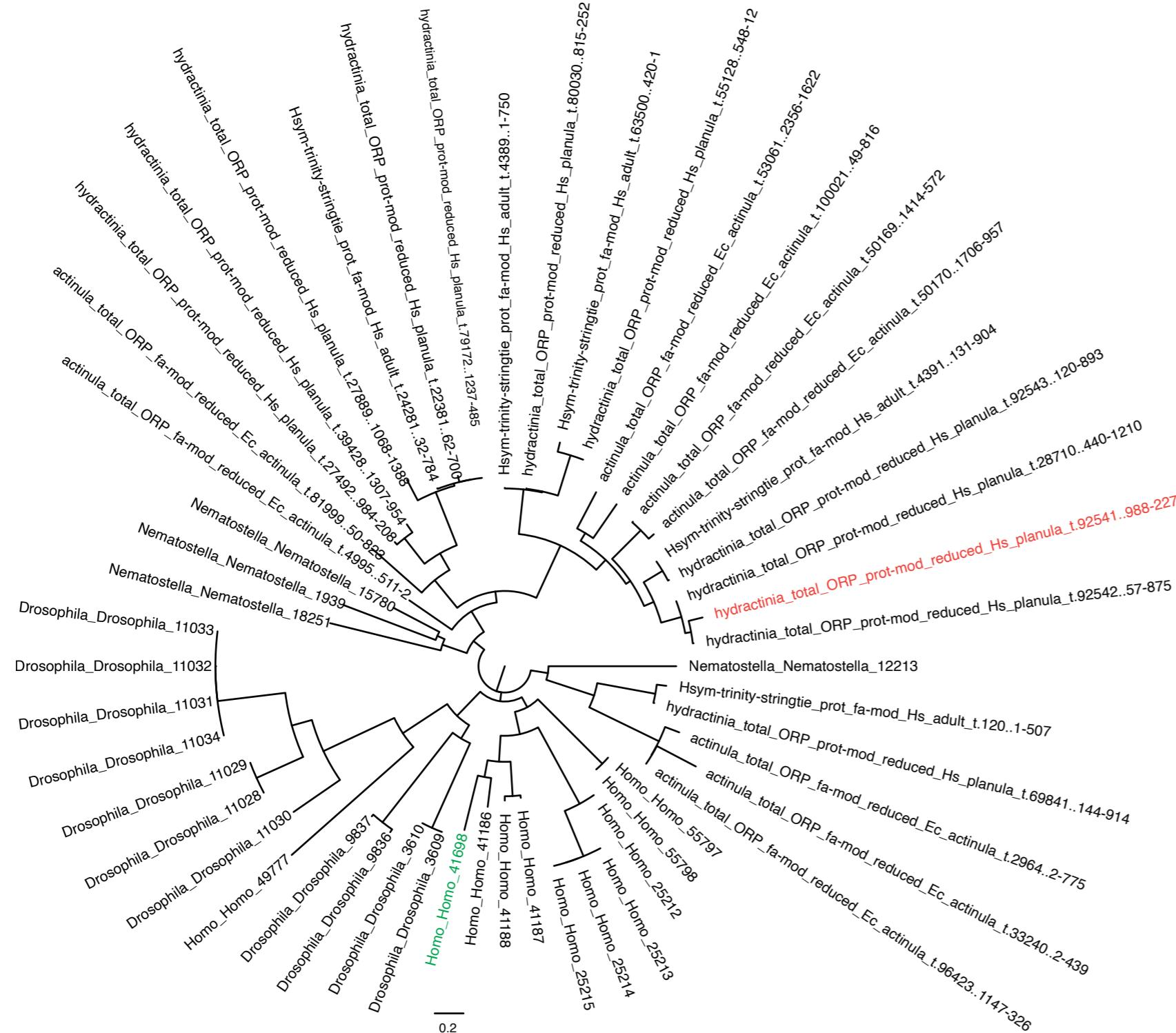

## CNGA3;CNGB3 OG0001196

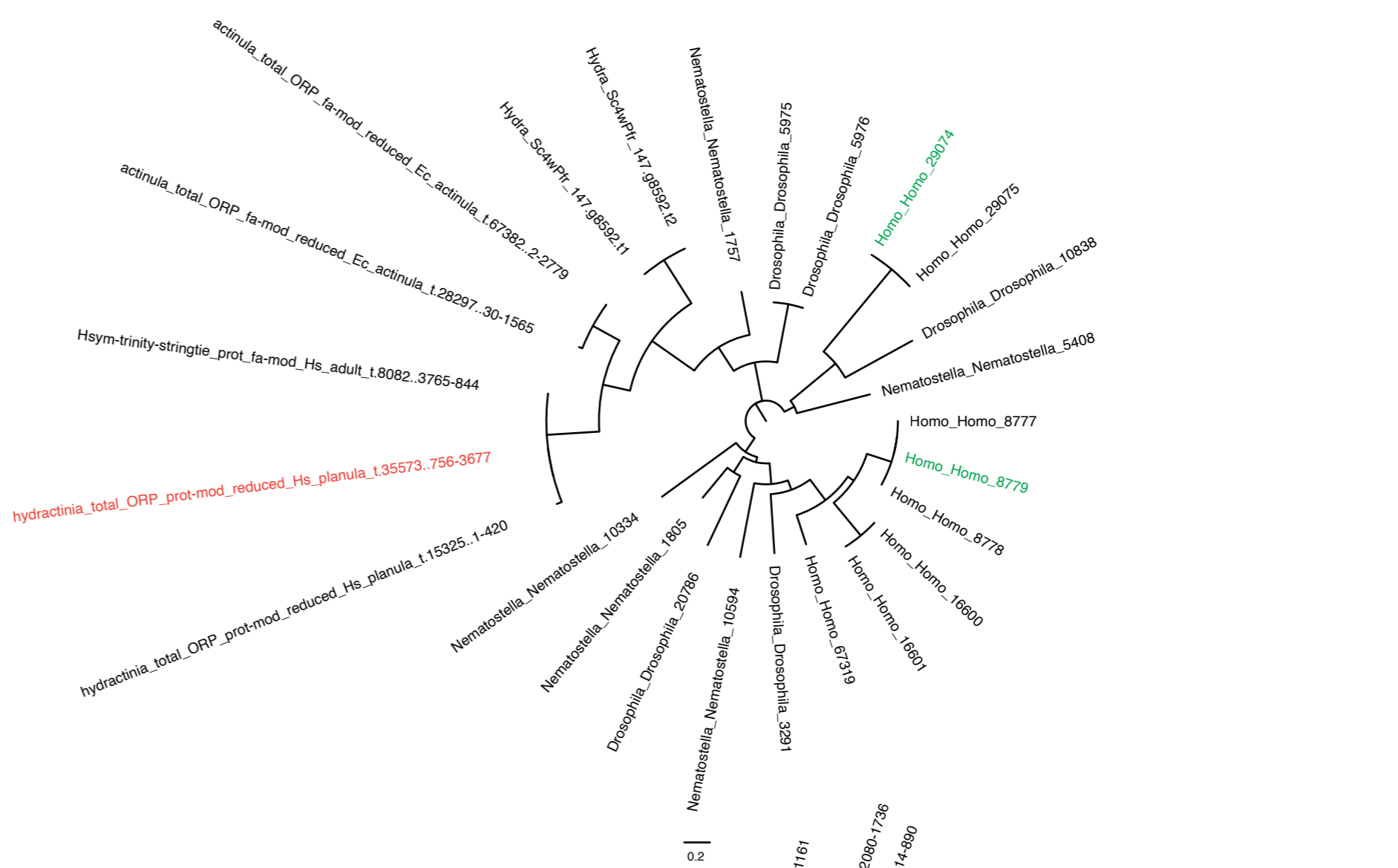

CRX; VSX; VSX2; RAX  
OG0000012

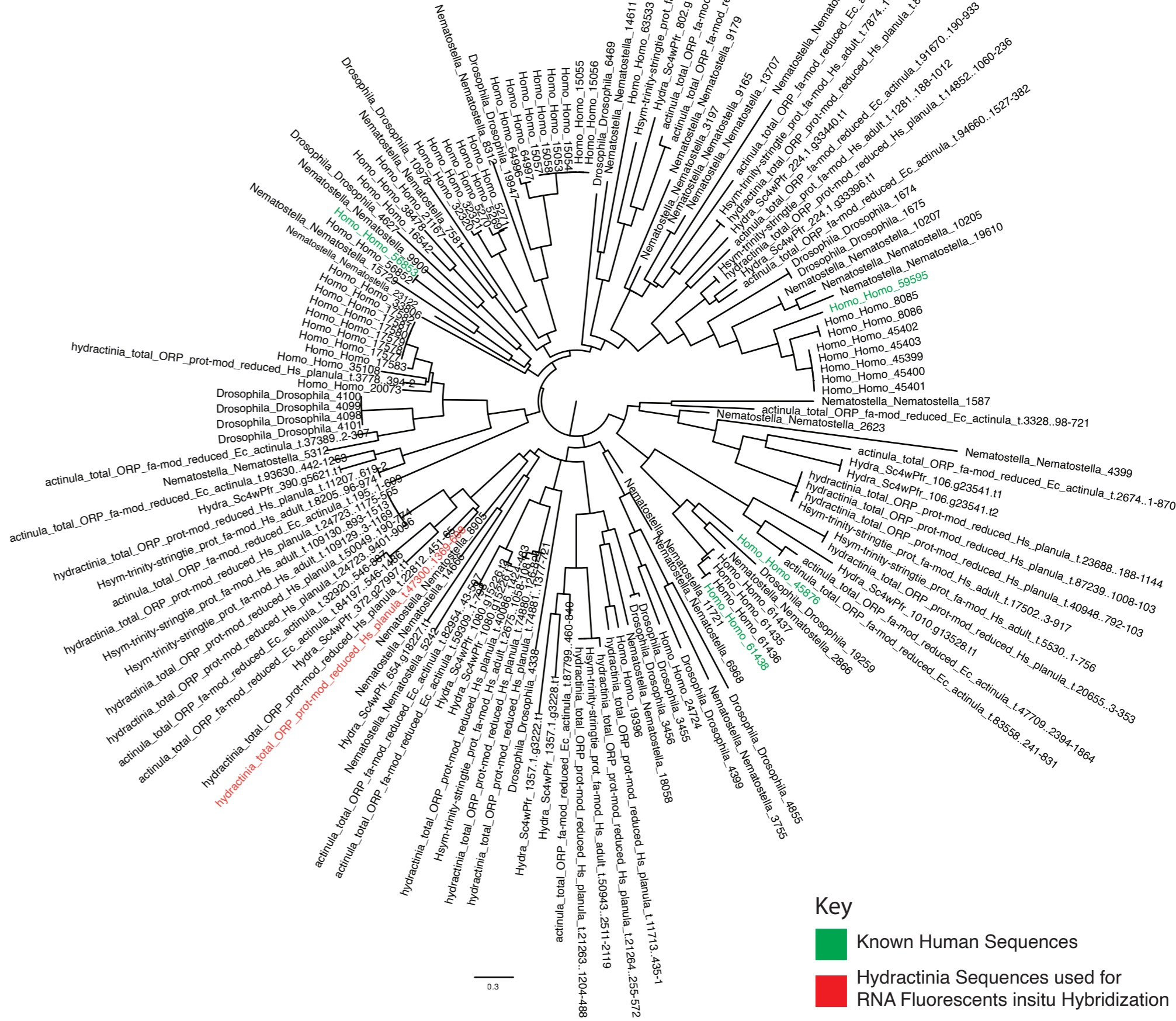

Supplement: Supplement 14 [file media-14.zip › Supp_Fig_6.pdf]
