# Supplementary material for: Phototactic preference and its genetic basis in the planulae of the colonial Hydrozoan Hydractinia symbiolongicarpus
Source: bioRxiv. 2024 Apr 1:2024.03.28.585045. Preprint. [Version 1] doi: 10.1101/2024.03.28.585045 (PMC11014542; doi:10.1101/2024.03.28.585045)
Supplement: Supplement 3 — Figure 3. Differential gene expression over development in the sensory perception of light stimulus gene set. (A) Differentially expressed genes over four days of development examining the sensory perception of light stimulus gene set derived from the GSEA (33). Each row is a single transcript labeled by the human gene symbols that are present in the same orthogroup as the planula transcript. High expression is yellow and low is light blue. Grey panels contain abbreviated descriptions of gene functions from UniProt (116). (B-D) STRING networks showing significantly differentially expressed genes across four days of development where (B) contains all DE genes from all four days, and (C) contains subnetworks of DE genes by day. STRING is a database of known and predicted protein-protein interactions (36). In each interaction map, line thickness indicates the strength of data support. Gene symbols are shaded by biological processes (Gene Ontology) or Annotated Keywords (UniProt). (D) Node and edge information for each network that includes the PPI enrichment p-values. PPI enrichment p-values double in days 3 and 4, consistent with the expression of phototaxis behavior. [file media-3.zip › FIg_3.pdf]

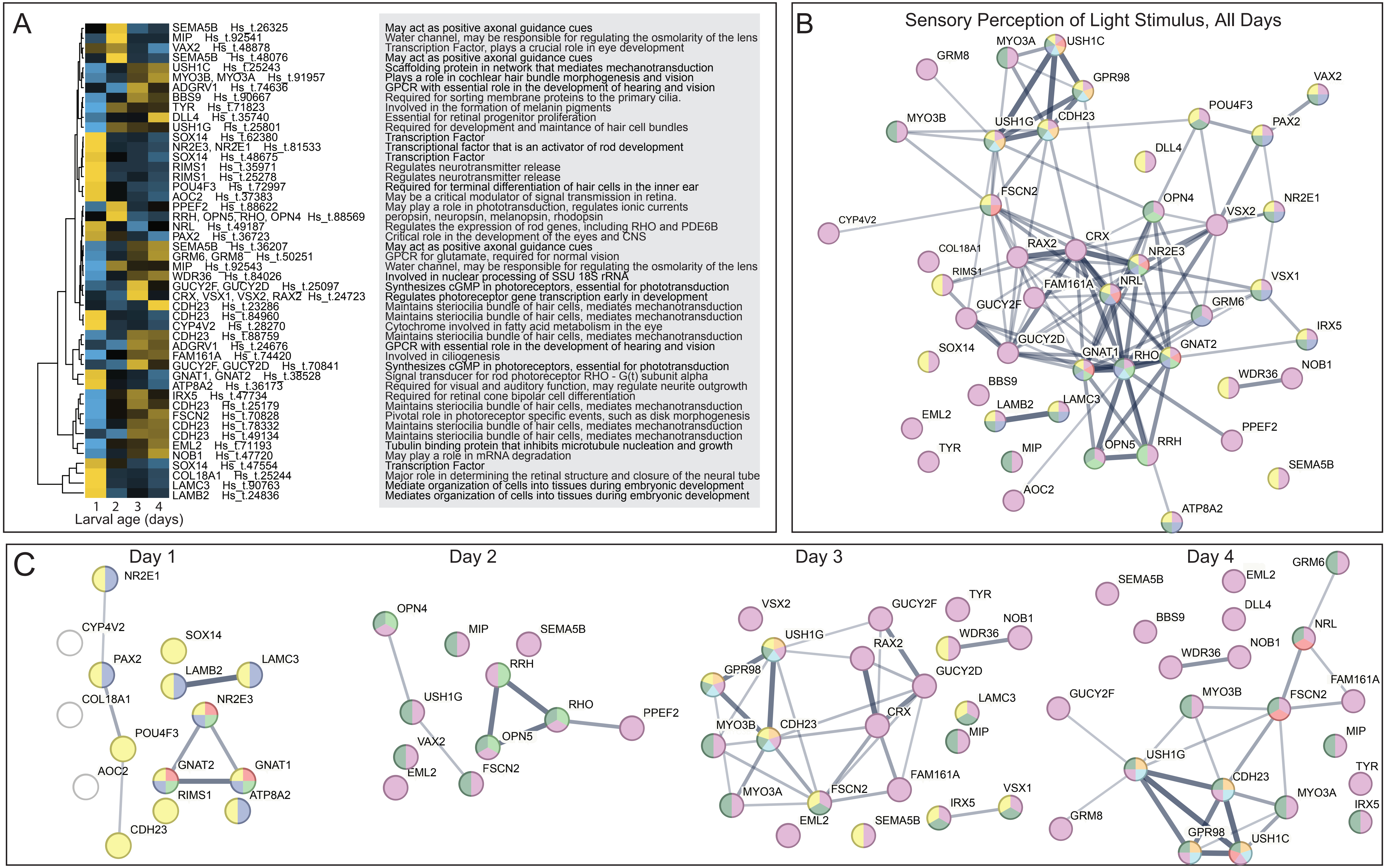

D Statistics on String Networks

	#of nodes	# of edges	Expected # of edges	PPI enrichment p-value
All Days	46	109	7	<1.0e-16***
Day 1	15	7	1	4.88e-06***
Day 2	11	6	0	5.46e-0.8***
Day 3	21	27	2	<1.0e-16***
Day 4	21	22	2	<1.0e-16***

Key

- Eye photoreceptor cell development
- Retina development
- Phototransduction
- Neurogenesis
- Sensory Perception of Light Stimulus
- Sensory Organ Development
- Photoreceptor cell maintenance
- Inner ear receptor cell stereocilium organization

Low High

Line thickness indicates the strength of data support
