# Supplementary material for: Phototactic preference and its genetic basis in the planulae of the colonial Hydrozoan Hydractinia symbiolongicarpus
Source: bioRxiv. 2024 Apr 1:2024.03.28.585045. Preprint. [Version 1] doi: 10.1101/2024.03.28.585045 (PMC11014542; doi:10.1101/2024.03.28.585045)
Supplement: Supplement 5 — Figure 5. Immunohistochemistry of the nervous system and FMRFamide expression in an H. symbiolongicarpus planula larvae. Immunohistochemistry staining of a Day 3 larva (72hpf) where red staining corresponds to acetylated alpha-tubulin of neural cells, magenta corresponds to RFamide, a neurotransmitter involved in relaying photosensory information, green corresponds to F-actin in contractile muscle, and blue corresponds to DAPI staining of nuclei. (A) Depicts the whole larva with different merges of the four channels. (B) Depicts the zoomed-in view of the aboral plexus which is the site of sensory integration and settlement. [file media-5.zip › Fig_5.pdf]

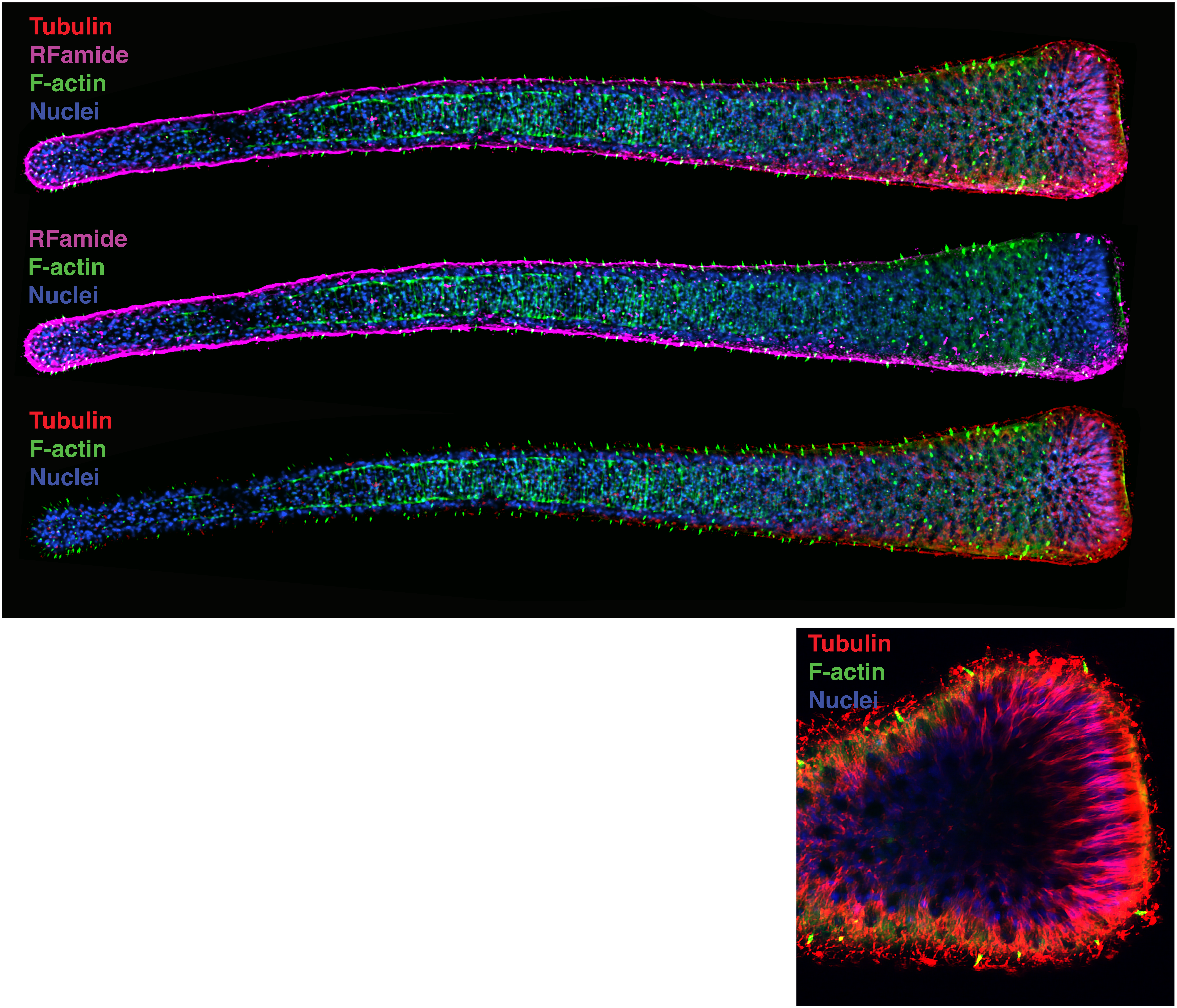
Tubulin
RFamide
F-actin
Nuclei
RFamide
F-actin
Nuclei
Tubulin
F-actin
Nuclei
Tubulin
F-actin
Nuclei
