# Supplementary material for: Phototactic preference and its genetic basis in the planulae of the colonial Hydrozoan Hydractinia symbiolongicarpus
Source: bioRxiv. 2024 Apr 1:2024.03.28.585045. Preprint. [Version 1] doi: 10.1101/2024.03.28.585045 (PMC11014542; doi:10.1101/2024.03.28.585045)
Supplement: Supplement 9 [file media-9.zip › Supp_Fig_1.pdf]

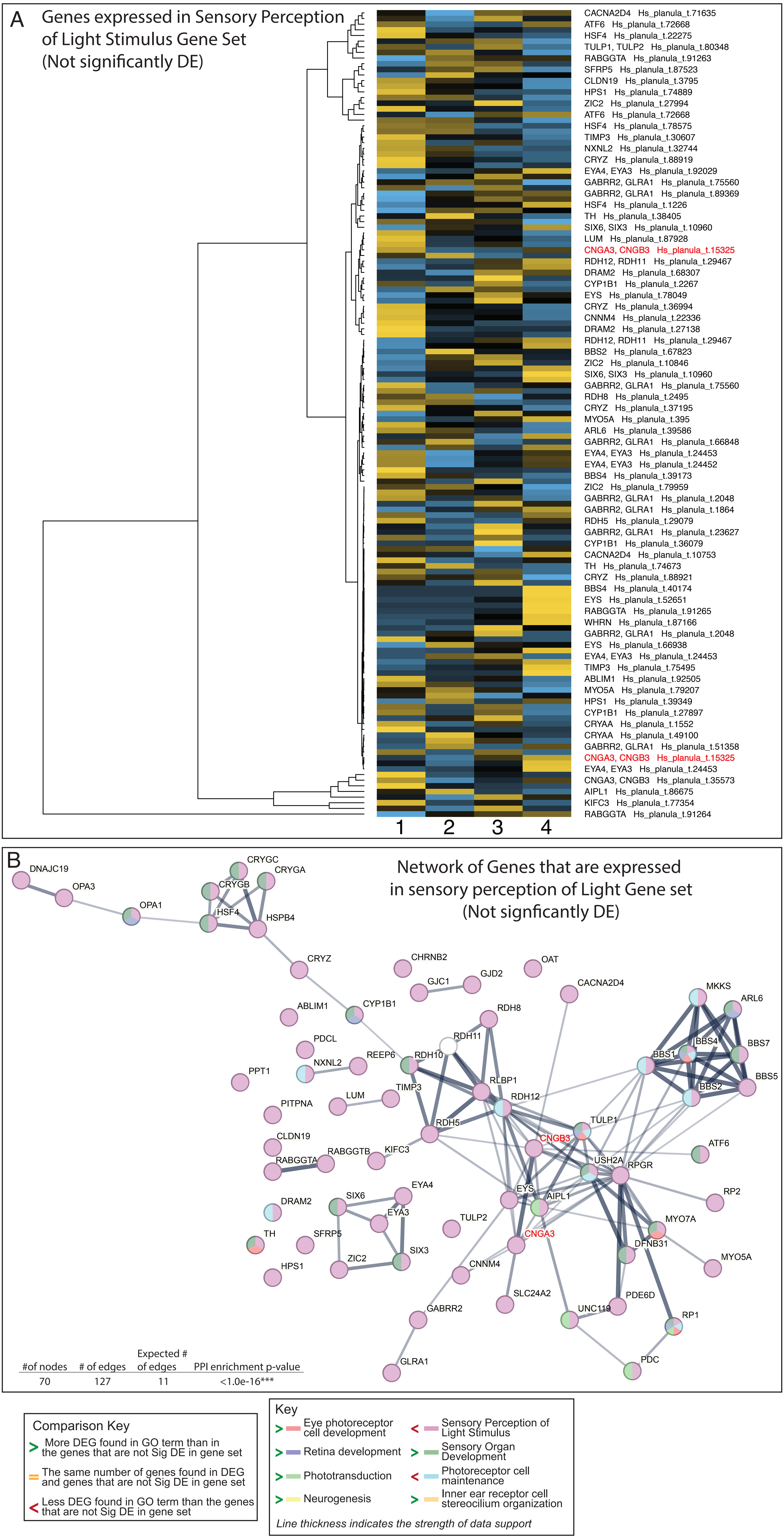
A
Genes expressed in Sensory Perception of Light Stimulus Gene Set (Not significantly DE)
CACNA2D4 Hs_planula_t.71635
ATF6 Hs_planula_t.72668
HSF4 Hs_planula_t.22275
TULP1, TULP2 Hs_planula_t.80348
RABGGTA Hs_planula_t.91263
SFRP5 Hs_planula_t.87523
CLDN19 Hs_planula_t.3795
HPS1 Hs_planula_t.74889
ZIC2 Hs_planula_t.27994
ATF6 Hs_planula_t.72668
HSF4 Hs_planula_t.78575
TIMP3 Hs_planula_t.30607
NXNL2 Hs_planula_t.32744
CRYZ Hs_planula_t.88919
EYA4, EYA3 Hs_planula_t.92029
GABRR2, GLRA1 Hs_planula_t.75560
GABRR2, GLRA1 Hs_planula_t.89369
HSF4 Hs_planula_t.1226
TH Hs_planula_t.38405
SIX6, SIX3 Hs_planula_t.10960
LUM Hs_planula_t.87928
CNGA3, CNGB3 Hs_planula_t.15325
RDH12, RDH11 Hs_planula_t.29467
DRAM2 Hs_planula_t.68307
CYP1B1 Hs_planula_t.2267
EYS Hs_planula_t.78049
CRYZ Hs_planula_t.36994
CNNM4 Hs_planula_t.22336
DRAM2 Hs_planula_t.27138
RDH12, RDH11 Hs_planula_t.29467
BBS2 Hs_planula_t.67823
ZIC2 Hs_planula_t.10846
SIX6, SIX3 Hs_planula_t.10960
GABRR2, GLRA1 Hs_planula_t.75560
RDH8 Hs_planula_t.2495
CRYZ Hs_planula_t.37195
MYO5A Hs_planula_t.395
ARL6 Hs_planula_t.39586
GABRR2, GLRA1 Hs_planula_t.66848
EYA4, EYA3 Hs_planula_t.24453
EYA4, EYA3 Hs_planula_t.24452
BBS4 Hs_planula_t.39173
ZIC2 Hs_planula_t.79959
GABRR2, GLRA1 Hs_planula_t.2048
GABRR2, GLRA1 Hs_planula_t.1864
RDH5 Hs_planula_t.29079
GABRR2, GLRA1 Hs_planula_t.23627
CYP1B1 Hs_planula_t.36079
CACNA2D4 Hs_planula_t.10753
TH Hs_planula_t.74673
CRYZ Hs_planula_t.88921
BBS4 Hs_planula_t.40174
EYS Hs_planula_t.52651
RABGGTA Hs_planula_t.91265
WHRN Hs_planula_t.87166
GABRR2, GLRA1 Hs_planula_t.2048
EYS Hs_planula_t.66938
EYA4, EYA3 Hs_planula_t.24453
TIMP3 Hs_planula_t.75495
ABLIM1 Hs_planula_t.92505
MYO5A Hs_planula_t.79207
HPS1 Hs_planula_t.39349
CYP1B1 Hs_planula_t.27897
CRYAA Hs_planula_t.1552
CRYAA Hs_planula_t.49100
GABRR2, GLRA1 Hs_planula_t.51358
CNGA3, CNGB3 Hs_planula_t.15325
EYA4, EYA3 Hs_planula_t.24453
CNGA3, CNGB3 Hs_planula_t.35573
AIPL1 Hs_planula_t.86675
KIFC3 Hs_planula_t.77354
RABGGTA Hs_planula_t.91264
1
2
3
4
B
Network of Genes that are expressed in sensory perception of Light Gene set (Not signficantly DE)
DNAJC19
OPA3
OPA1
CRYGC
CRYGA
CRYGB
HSF4
HSPB4
CRYZ
CHRNB2
OAT
GJD2
GJC1
CACNA2D4
MKKS
ARL6
ABLIM1
CYP1B1
RDH8
PDCL
RDH11
BBS4
BBS7
REEP6
RDH10
BBS1
NXNL2
PPT1
RLBP1
BBS5
TIMP3
BBS2
RDH12
LUM
PITPNA
RDH5
TULP1
CLDN19
CNGB3
ATF6
RABGGTB
KIFC3
RABGGTA
USH2A
RPGR
EYA4
DRAM2
SIX6
EYS
AIPL1
RP2
EYA3
TULP2
MYO7A
TH
SFRP5
CNGA3
SIX3
DFNB31
ZIC2
HPS1
CNNM4
MYO5A
SLC24A2
PDE6D
GABRR2
UNC119
RP1
GLRA1
PDC
#of nodes | # of edges | Expected # of edges | PPI enrichment p-value
70 | 127 | 11 | <1.0e-16***
Comparison Key
> More DEG found in GO term than in the genes that are not Sig DE in gene set
= The same number of genes found in DEG and genes that are not Sig DE in gene set
< Less DEG found in GO term than the genes that are not Sig DE in gene set
Key
> Eye photoreceptor cell development
< Sensory Perception of Light Stimulus
> Retina development
> Sensory Organ Development
> Phototransduction
< Photoreceptor cell maintenance
> Neurogenesis
> Inner ear receptor cell stereocilium organization
Line thickness indicates the strength of data support
