# Supplementary material for: Phototactic preference and its genetic basis in the planulae of the colonial Hydrozoan Hydractinia symbiolongicarpus
Source: bioRxiv. 2024 Apr 1:2024.03.28.585045. Preprint. [Version 1] doi: 10.1101/2024.03.28.585045 (PMC11014542; doi:10.1101/2024.03.28.585045)
Supplement: Supplement 11 [file media-11.zip › Supp_Fig_3.pdf]

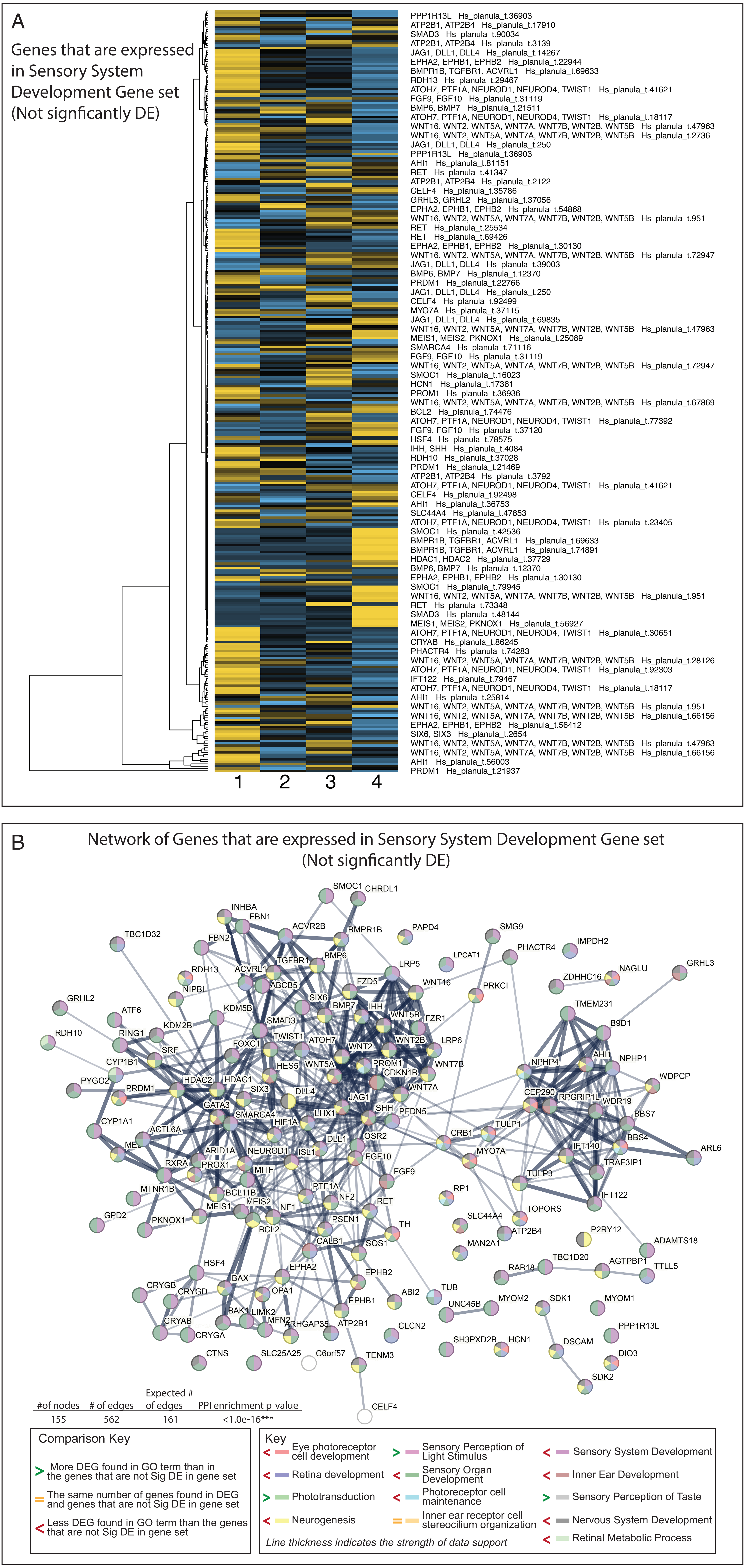

A
Genes that are expressed in Sensory System Development Gene set (Not signficantly DE)
PPP1R13L Hs_planula_t.36903
ATP2B1, ATP2B4 Hs_planula_t.17910
SMAD3 Hs_planula_t.90034
ATP2B1, ATP2B4 Hs_planula_t.3139
JAG1, DLL1, DLL4 Hs_planula_t.14267
EPHA2, EPHB1, EPHB2 Hs_planula_t.22944
BMPR1B, TGFBR1, ACVRL1 Hs_planula_t.69633
RDH13 Hs_planula_t.29467
ATOH7, PTF1A, NEUROD1, NEUROD4, TWIST1 Hs_planula_t.41621
FGF9, FGF10 Hs_planula_t.31119
BMP6, BMP7 Hs_planula_t.21511
ATOH7, PTF1A, NEUROD1, NEUROD4, TWIST1 Hs_planula_t.18117
WNT16, WNT2, WNT5A, WNT7A, WNT7B, WNT2B, WNT5B Hs_planula_t.47963
WNT16, WNT2, WNT5A, WNT7A, WNT7B, WNT2B, WNT5B Hs_planula_t.2736
JAG1, DLL1, DLL4 Hs_planula_t.250
PPP1R13L Hs_planula_t.36903
AHI1 Hs_planula_t.81151
RET Hs_planula_t.41347
ATP2B1, ATP2B4 Hs_planula_t.2122
CELF4 Hs_planula_t.35786
GRHL3, GRHL2 Hs_planula_t.37056
EPHA2, EPHB1, EPHB2 Hs_planula_t.54868
WNT16, WNT2, WNT5A, WNT7A, WNT7B, WNT2B, WNT5B Hs_planula_t.951
RET Hs_planula_t.25534
RET Hs_planula_t.69426
EPHA2, EPHB1, EPHB2 Hs_planula_t.30130
WNT16, WNT2, WNT5A, WNT7A, WNT7B, WNT2B, WNT5B Hs_planula_t.72947
JAG1, DLL1, DLL4 Hs_planula_t.39003
BMP6, BMP7 Hs_planula_t.12370
PRDM1 Hs_planula_t.22766
JAG1, DLL1, DLL4 Hs_planula_t.250
CELF4 Hs_planula_t.92499
MYO7A Hs_planula_t.37115
JAG1, DLL1, DLL4 Hs_planula_t.69835
WNT16, WNT2, WNT5A, WNT7A, WNT7B, WNT2B, WNT5B Hs_planula_t.47963
MEIS1, MEIS2, PKNOX1 Hs_planula_t.25089
SMARCA4 Hs_planula_t.71116
FGF9, FGF10 Hs_planula_t.31119
WNT16, WNT2, WNT5A, WNT7A, WNT7B, WNT2B, WNT5B Hs_planula_t.72947
SMOC1 Hs_planula_t.16023
HCN1 Hs_planula_t.17361
PROM1 Hs_planula_t.36936
WNT16, WNT2, WNT5A, WNT7A, WNT7B, WNT2B, WNT5B Hs_planula_t.67869
BCL2 Hs_planula_t.74476
ATOH7, PTF1A, NEUROD1, NEUROD4, TWIST1 Hs_planula_t.77392
FGF9, FGF10 Hs_planula_t.37120
HSF4 Hs_planula_t.78575
IHH, SHH Hs_planula_t.4084
RDH10 Hs_planula_t.37028
PRDM1 Hs_planula_t.21469
ATP2B1, ATP2B4 Hs_planula_t.3792
ATOH7, PTF1A, NEUROD1, NEUROD4, TWIST1 Hs_planula_t.41621
CELF4 Hs_planula_t.92498
AHI1 Hs_planula_t.36753
SLC44A4 Hs_planula_t.47853
ATOH7, PTF1A, NEUROD1, NEUROD4, TWIST1 Hs_planula_t.23405
SMOC1 Hs_planula_t.42536
BMPR1B, TGFBR1, ACVRL1 Hs_planula_t.69633
BMPR1B, TGFBR1, ACVRL1 Hs_planula_t.74891
HDAC1, HDAC2 Hs_planula_t.37729
BMP6, BMP7 Hs_planula_t.12370
EPHA2, EPHB1, EPHB2 Hs_planula_t.30130
SMOC1 Hs_planula_t.79945
WNT16, WNT2, WNT5A, WNT7A, WNT7B, WNT2B, WNT5B Hs_planula_t.951
RET Hs_planula_t.73348
SMAD3 Hs_planula_t.48144
MEIS1, MEIS2, PKNOX1 Hs_planula_t.56927
ATOH7, PTF1A, NEUROD1, NEUROD4, TWIST1 Hs_planula_t.30651
CRYAB Hs_planula_t.86245
PHACTR4 Hs_planula_t.74283
WNT16, WNT2, WNT5A, WNT7A, WNT7B, WNT2B, WNT5B Hs_planula_t.28126
ATOH7, PTF1A, NEUROD1, NEUROD4, TWIST1 Hs_planula_t.92303
IFT122 Hs_planula_t.79467
ATOH7, PTF1A, NEUROD1, NEUROD4, TWIST1 Hs_planula_t.18117
AHI1 Hs_planula_t.25814
WNT16, WNT2, WNT5A, WNT7A, WNT7B, WNT2B, WNT5B Hs_planula_t.951
WNT16, WNT2, WNT5A, WNT7A, WNT7B, WNT2B, WNT5B Hs_planula_t.66156
EPHA2, EPHB1, EPHB2 Hs_planula_t.56412
SIX6, SIX3 Hs_planula_t.2654
WNT16, WNT2, WNT5A, WNT7A, WNT7B, WNT2B, WNT5B Hs_planula_t.47963
WNT16, WNT2, WNT5A, WNT7A, WNT7B, WNT2B, WNT5B Hs_planula_t.66156
AHI1 Hs_planula_t.56003
PRDM1 Hs_planula_t.21937
1 2 3 4
B
Network of Genes that are expressed in Sensory System Development Gene set (Not signficantly DE)
#of nodes 155
# of edges 562
Expected # of edges 161
PPI enrichment p-value <1.0e-16***
Comparison Key
> More DEG found in GO term than in the genes that are not Sig DE in gene set
= The same number of genes found in DEG and genes that are not Sig DE in gene set
< Less DEG found in GO term than the genes that are not Sig DE in gene set
Key
< Eye photoreceptor cell development
< Retina development
> Phototransduction
< Neurogenesis
> Sensory Perception of Light Stimulus
< Sensory Organ Development
< Photoreceptor cell maintenance
= Inner ear receptor cell stereocilium organization
< Sensory System Development
< Inner Ear Development
> Sensory Perception of Taste
< Nervous System Development
< Retinal Metabolic Process
Line thickness indicates the strength of data support
