# Supplementary material for: Phototactic preference and its genetic basis in the planulae of the colonial Hydrozoan Hydractinia symbiolongicarpus
Source: bioRxiv. 2024 Apr 1:2024.03.28.585045. Preprint. [Version 1] doi: 10.1101/2024.03.28.585045 (PMC11014542; doi:10.1101/2024.03.28.585045)
Supplement: Supplement 12 [file media-12.zip › Supp_Fig_4.pdf]

Network of Genes that are Not Expressed in Sensory System Development Gene set

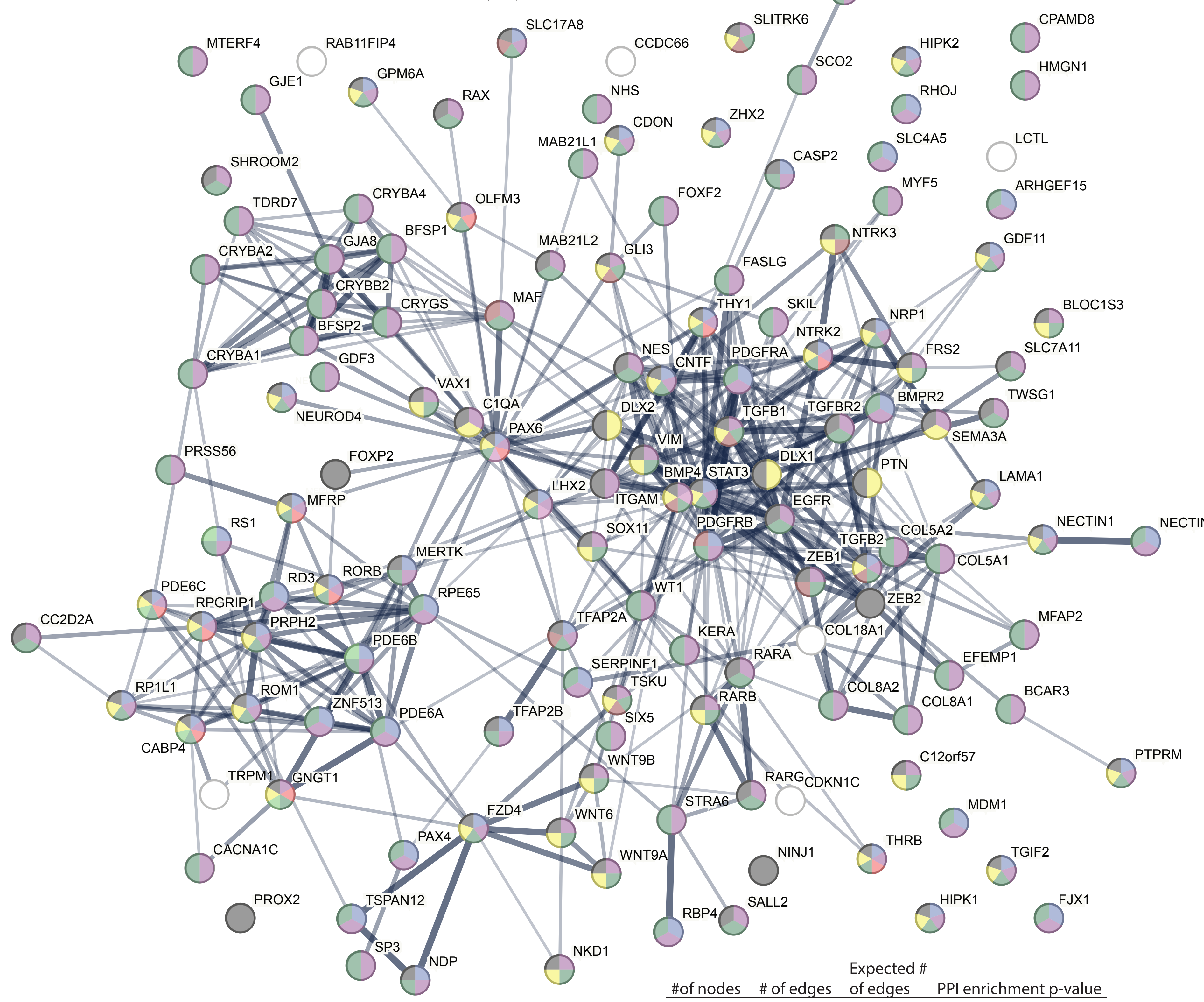

#of nodes	# of edges	Expected # of edges	PPI enrichment p-value
140	438	105	<1.0e-16***

Key

- Eye photoreceptor cell development
- Neurogenesis
- Photoreceptor cell maintenance
- Inner Ear Development
- Nervous System Development
- Retina development
- Sensory Perception of Light Stimulus
- Inner ear receptor cell stereocilium organization
- Sensory Perception of Taste
- Retinal Metabolic Process
- Phototransduction
- Sensory Organ Development
- Sensory System Development

Line thickness indicates the strength of data support
