# Supplementary material for: Phototactic preference and its genetic basis in the planulae of the colonial Hydrozoan Hydractinia symbiolongicarpus
Source: bioRxiv. 2024 Apr 1:2024.03.28.585045. Preprint. [Version 1] doi: 10.1101/2024.03.28.585045 (PMC11014542; doi:10.1101/2024.03.28.585045)
Supplement: Supplement 13 [file media-13.zip › Supp_Fig_5.pdf]

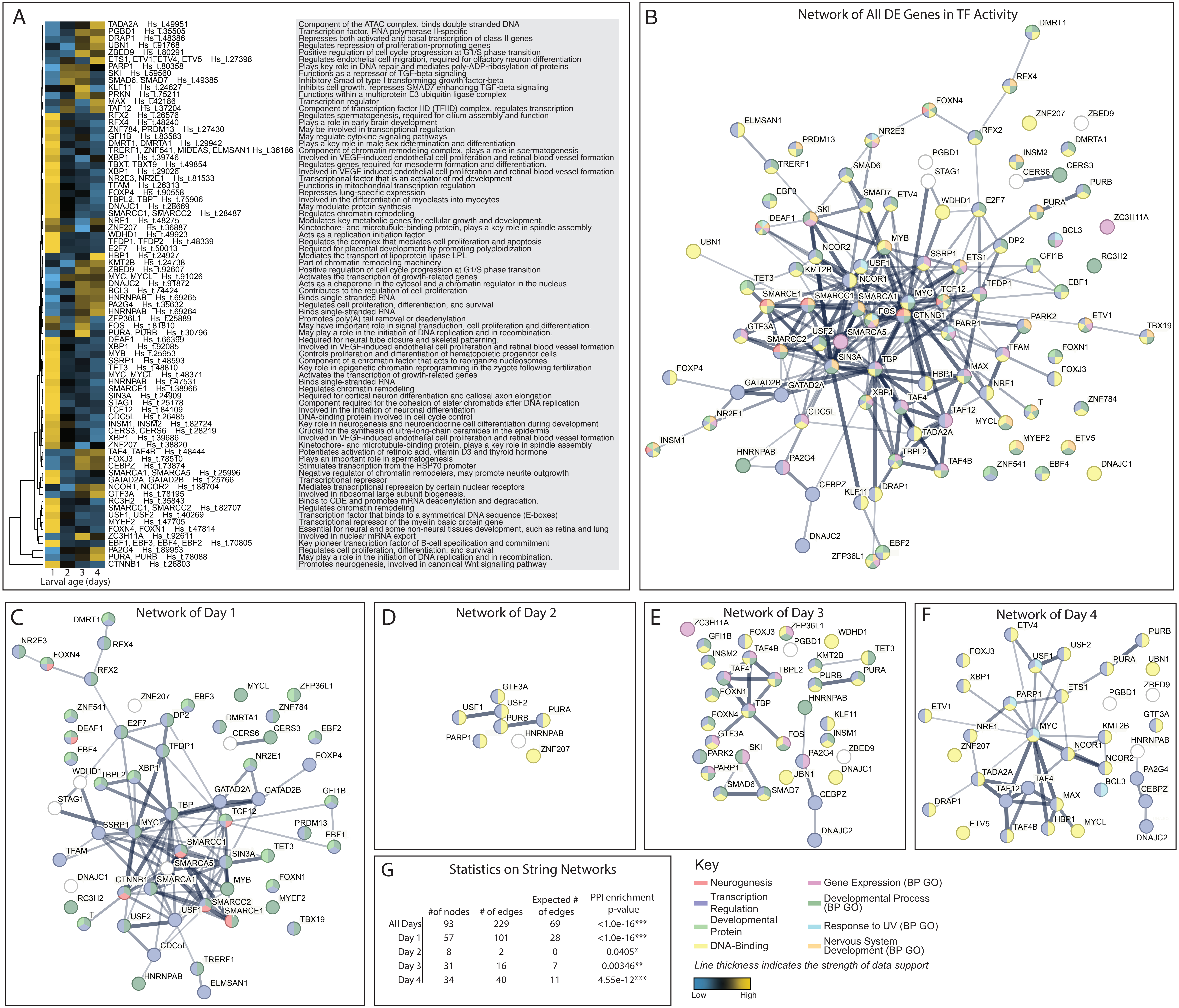

	#of nodes	# of edges	Expected # of edges	PPI enrichment p-value
All Days	93	229	69	<1.0e-16***
Day 1	57	101	28	<1.0e-16***
Day 2	8	2	0	0.0405*
Day 3	31	16	7	0.00346**
Day 4	34	40	11	4.55e-12***
