# Supplementary material for: Phototactic preference and its genetic basis in the planulae of the colonial Hydrozoan Hydractinia symbiolongicarpus
Source: bioRxiv. 2024 Apr 1:2024.03.28.585045. Preprint. [Version 1] doi: 10.1101/2024.03.28.585045 (PMC11014542; doi:10.1101/2024.03.28.585045)
Supplement: Supplement 7 — Figure 7. Colocalization of photosensory transcripts in the aboral region of the planula. Multiplexed RNA Fluorescent in situ hybridization (FISH) of photosensory transcripts including: cnidopsin (green, Hs_t.88569), CNG (red, Hs_t.35573), CRX (magenta, Hs_t.47300), and DAPI (Blue). (A) Merged Z-Stack of all channels. (B) Each combination of individual transcript expression in single optical slice of a whole mount specimen. (C-I) Higher magnification of the aboral region with a focus on the bipolar sensory neurons of the aboral neural plexus. Cnidopsin, CNG ion channels, and CRX strongly localize to the ciliated region of bipolar sensory neurons. [file Fig_7.pdf › Fig_7.pdf]

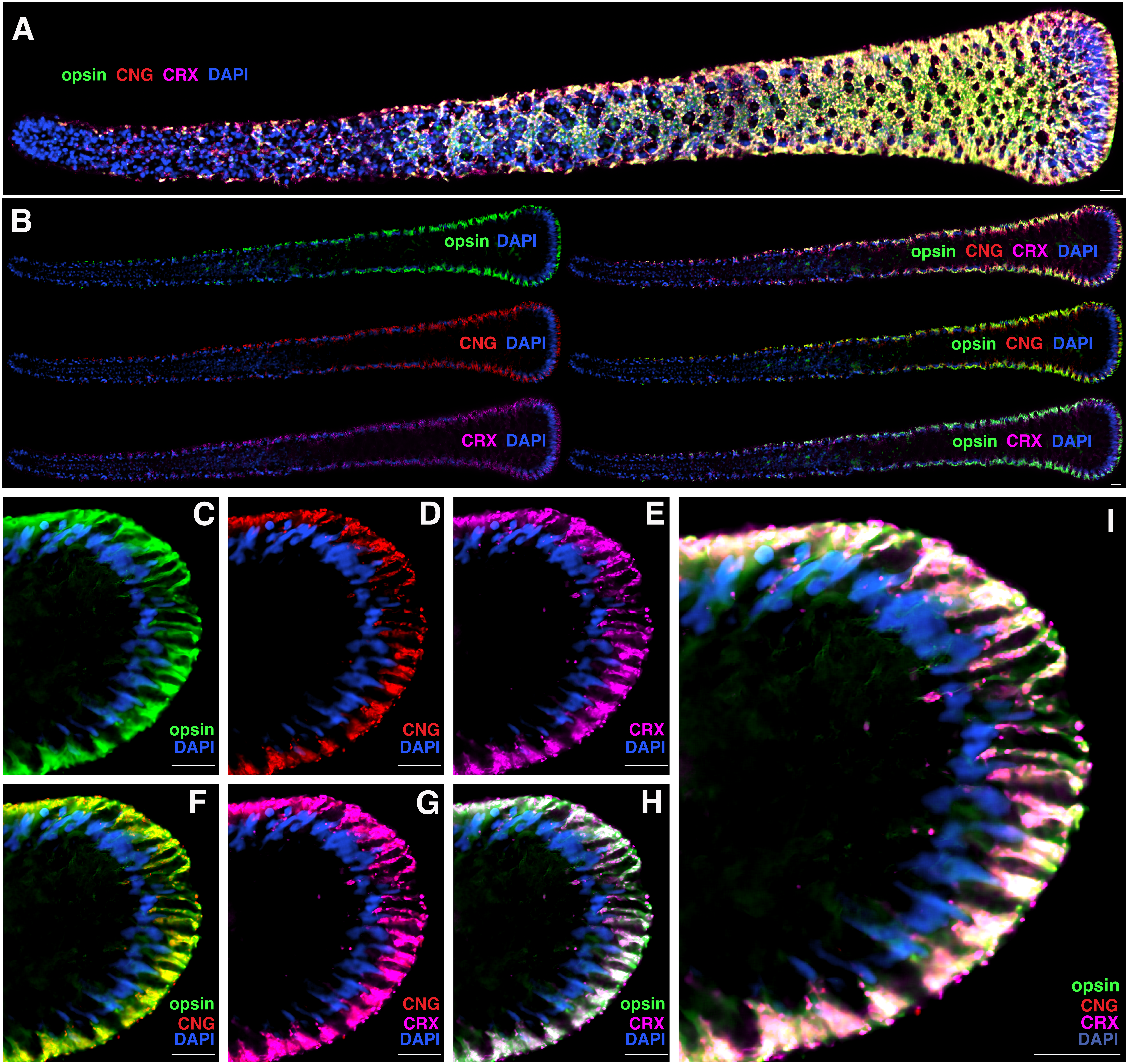

A
opsin CNG CRX DAPI
B
opsin DAPI
opsin CNG CRX DAPI
CNG DAPI
opsin CNG DAPI
CRX DAPI
opsin CRX DAPI
C
opsin
DAPI
D
CNG
DAPI
E
CRX
DAPI
F
opsin
CNG
DAPI
G
CNG
CRX
DAPI
H
opsin
CRX
DAPI
I
opsin
CNG
CRX
DAPI
