# Supplementary material for: Phototactic preference and its genetic basis in the planulae of the colonial Hydrozoan Hydractinia symbiolongicarpus
Source: bioRxiv. 2024 Apr 1:2024.03.28.585045. Preprint. [Version 1] doi: 10.1101/2024.03.28.585045 (PMC11014542; doi:10.1101/2024.03.28.585045)
Supplement: Supplement 1 — Figure 1. The wavelength-dependent phototactic response of H. symbiolongicarpus planulae. (A) We measured phototaxis in day 3 (72hpf) H. symbiolongicarpus planulae using heatless LEDs of known wavelengths in behavioral arenas kept in darkness. Planulae were placed in petri dishes above the LEDs and spatially randomized when ‘before’ photographs were taken. Light of a specific wavelength was then applied for eight hours and an “after” photograph was taken. Images from one replicate are shown. (B) For each light condition, we compared the number of respondent (phototactic) larvae to the number of non-respondent larvae using the Welch two-sample t-test. Larvae of H. symbiolongicarpus are strongly sensitive to green and blue light, but no significant difference was observed between the number of respondent and non-respondent planulae in red trials. At least 5 separate trials were conducted for each light condition. ** p ≤ 0.01; *** p ≤ 0.001 [file media-1.zip › Fig_1.pdf]

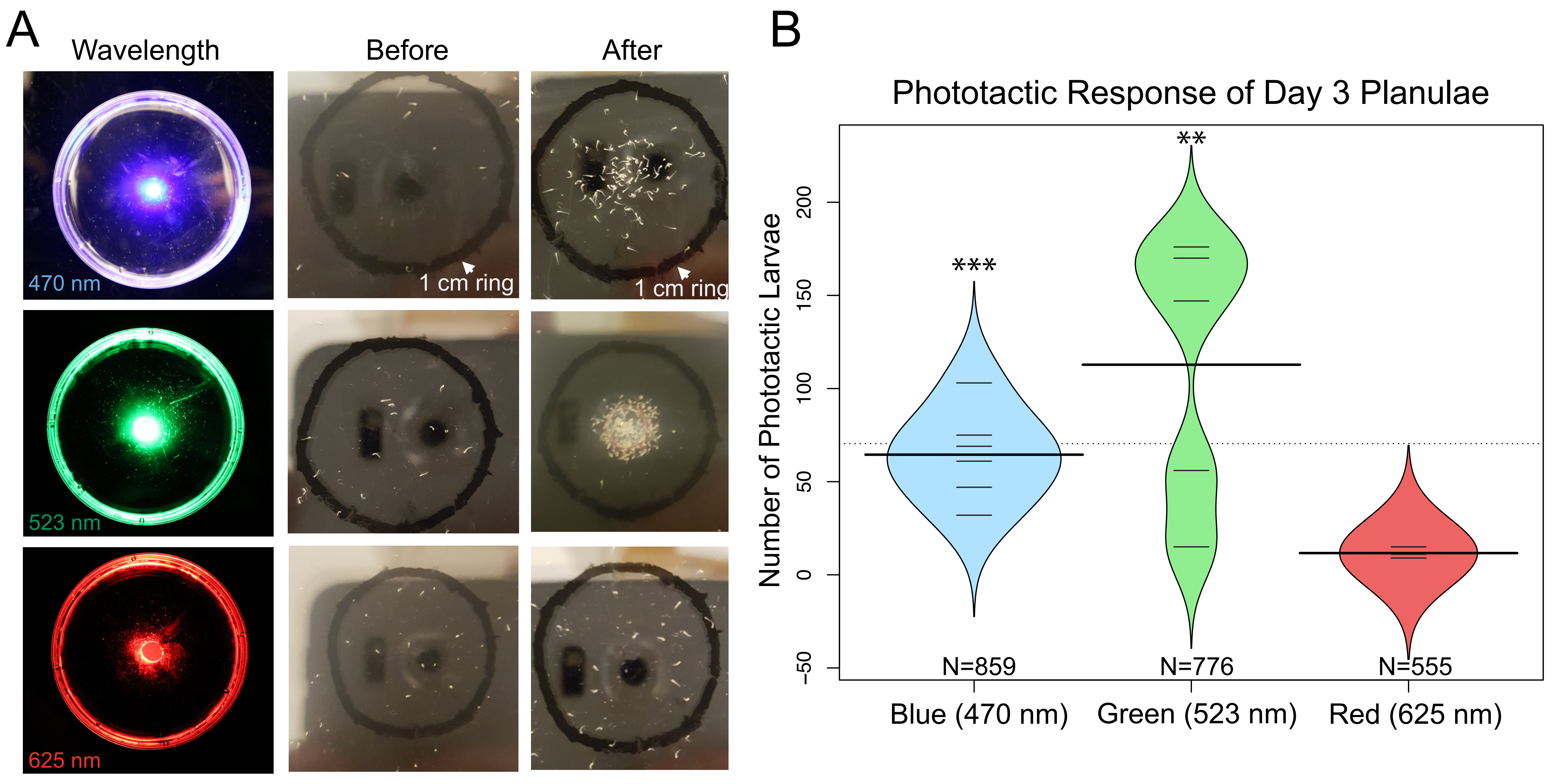
A
Wavelength
Before
After
470 nm
1 cm ring
1 cm ring
523 nm
625 nm
B
Phototactic Response of Day 3 Planulae
Number of Phototactic Larvae
200
150
100
50
0
-50
***
**
N=859
N=776
N=555
Blue (470 nm)
Green (523 nm)
Red (625 nm)
